# *In vitro* and *in vivo* characterization of DNA delivery using recombinant *Lactococcus lactis* expressing a mutated form of *L*. *monocytogenes* Internalin A

**DOI:** 10.1186/1471-2180-12-299

**Published:** 2012-12-19

**Authors:** Marcela de Azevedo, Jurgen Karczewski, François Lefévre, Vasco Azevedo, Anderson Miyoshi, Jerry M Wells, Philippe Langella, Jean-Marc Chatel

**Affiliations:** 1 INRA, UMR1319 Micalis, Commensals and Probiotics-Host Interactions Laboratory, Jouy-en-Josas, France; 2 AgroParisTech, UMR Micalis, F-78350, Jouy-en-Josas, France; 3 Host Microbe Interactomics, Wageningen University, Wageningen, The Netherlands; 4 Laboratorio de Genética Celular e Molecular, ICB, UFMG, Minas Gerais, Brazil; 5 INRA, VIM, Jouy-en-Josas, France

**Keywords:** Lactococcus lactis, Listeria monocytogenes, Mutated internalin A, Internalization, DNA delivery

## Abstract

**Background:**

The use of food-grade Lactic Acid Bacteria (LAB) as DNA delivery vehicles represents an attractive strategy to deliver DNA vaccines at the mucosal surfaces as they are generally regarded as safe (GRAS). We previously showed that either native *Lactococcus lactis* (LL) or recombinant invasive LL expressing Fibronectin Binding Protein A of *Staphylococcus aureus* (LL-FnBPA+) or Internalin A of *Listeria monocytogenes* (LL-InlA+), were able to deliver and trigger DNA expression by epithelial cells, either *in vitro* or *in vivo*. InlA does not bind to its receptor, the murine E-cadherin, thus limiting the use of LL-InlA+ in *in vivo* murine models. Moreover, FnBPA binds to its receptors, integrins, via fibronectin introducing another limiting factor. In order to avoid the limitations of LL-InlA+ and LL-FnBPA+, a new *L*. *lactis* strain was engineered to produce a previously described mutated form of InlA (LL-mInlA+) allowing the binding of mInlA on murine E-cadherin.

**Results:**

After showing the expression of mInLA at the surface of LL-mInlA+ strain, *in vitro* gentamycin survival assay in Caco-2 cells showed that LL-mInlA+ is 1000 times more invasive than LL. LL-mInlA+ invasivity was also validated by fluorescence microscopy. LL and LL-mInlA+ were transformed with pValacBLG, a plasmid containing the cDNA of bovine β-Lactoglobulin (BLG), resulting in strains LL-BLG and LL-mInlA+BLG. The plasmid transfer *in vitro* using LL-mInlA+BLG was increased 10 times compared to LL-BLG. Moreover, the number of mice producing BLG in isolated enterocytes after oral administration of LL-mInlA+BLG *in vivo* was slightly higher than after oral administration of LL-BLG.

**Conclusions:**

We confirmed in this study that the production of mInlA at the surface of *L*. *lactis* is a promising strategy for plasmid transfer *in vitro* and *in vivo*.

## Background

DNA vaccination has gained a lot of attention since its ability to induce long-lasting humoral and cellular immune responses against an encoded antigen was discovered [1]. In addition, DNA vaccination poses no danger of integration into host cellular DNA thereby raising its safety profile [2-4]. DNA vaccines can be easily isolated to high purity, encode multiple antigens, and possess inherent adjuvant activity due to the presence of unmethylated CpG motifs that are recognized in mammals by TLR9 [5]. So called purified “Naked” DNA vaccination was shown to be highly efficient in rodents and mice, but not in larger animals and humans [6]. Consequently, it is very important to optimize DNA vaccine vectors and develop a delivery system to facilitate cellular uptake and enhance gene transfer efficiency and expression *in situ*[7].

Several strategies have been explored to protect plasmids from degradation, facilitating DNA uptake by phagocytic Antigen Presenting Cells (APCs) and thereby enhancing their immunological properties. This includes delivery technologies based on encapsulation into synthetic particles (cationic liposomes or polymers) or the use of viral vectors [7,8]. Despite their potential, some limitations and safety issues still remain which can restrict the application of gene therapy - e.g. the complexity of producing liposomes and their limited packaging capacity [9]. Additionally, it was shown that some viral vectors have the capacity to randomly integrate their genetic material into the host genome causing insertional mutagenesis of a cellular oncogene, leading to tumour formation [10].

The use of bacteria as delivery vehicles for DNA vaccination has emerged as an interesting alternative to overcome many of the problems associated with viral or liposomal delivery [11]. W. Schaffner was the first to observe genetic material transfer from bacteria to mammalian cells [12]. Since then, bacteria have been extensively exploited as vaccine delivery vehicles for vaccination against bacterial and viral pathogens as well as cancer immunotherapy [13-15]. The use of bacteria for mucosal delivery of DNA vaccines may be advantageous due to their potential to elicit secretory IgA responses as well as systemic immunity, when compared to conventional parenteral immunization [16]. Furthermore, bacterial carriers can increase and expand the magnitude of immune responses against the vector-encoded antigen due to the natural presence of Pathogen-Associated Molecular Patterns (PAMPs) that bind to Toll-like receptors (TLRs) and activate immune cells [5].

Presently, attenuated pathogens such as *Salmonella*, *Shigella*, *Listeria*, *Yersinia*, as well as, non-pathogenic *Escherichia coli* have been used as experimental live delivery systems [17,18]. An advantage of using attenuated pathogens as DNA vaccine vehicles is that they possess mechanisms to adhere or invade host cells with a negligible risk of reversion to a virulent strain via gene transfer or mutation. However, a potential concern is the risk of increased virulence in young or immunocompromised individuals.

The use of food-grade lactic acid bacteria (LAB) as DNA delivery vehicle represents an alternative and attractive strategy to deliver DNA vaccines at the mucosal surfaces (ref review by 19 and 20). The dietary group of LAB, including *Lactococcus lactis* and many species of *Lactobacillus*, is generally regarded as safe (GRAS) organisms of which some are intestinal commensals of humans. Indeed, it has been extensively demonstrated that these bacteria are able to deliver a range of vaccine and therapeutic molecules for applications in allergic, infectious or gastrointestinal diseases [19,21,22]. A relatively new development, however, is their use as a vehicle for genetic immunization [23]. Previous experiments performed by our group showed that either native *L*. *lactis* (LL) or recombinant invasive LL expressing Fibronectin Binding Protein A (LL-FnBPA+) of *Staphylococcus aureus* or Internalin A (InlA) of *Listeria monocytogenes* (LL-InlA+) [24,25], were able to deliver DNA in epithelial cells both *in vitro* and *in vivo*, demonstrating potential as gene transfer vehicles [24-27]. However InlA does not bind to its murine receptor, E-cadherin, thus limiting the use of LL-InlA+ in *in vivo* murine model. On the other hand, FnBPA requires an adequate local concentration of fibronectin to bind to its receptors, integrins [28,29].

In order to avoid the limitations of InlA and FnBPA and improve our knowledge on the key steps by which the DNA is transferred to mammalian cells using *L*. *lactis*, LL was engineered to express a mutated form of Internalin A (mInlA; Ser192Asn and Tyr369Ser) that increased binding affinity to murine and human E-cadherin [30,31] thus allowing for *in vivo* experiments in conventional mice. Herein, we describe the construction and characterization of this novel *L*. *lactis* strain as a DNA delivery vector, using cow’s milk β-lactoglobulin (BLG) allergen, to measure DNA transfer to intestinal epithelial cells (IECs) *in vitro* and *in vivo*.

Overall, the production of mInLA+at the surface of *Lactococcus lactis* increased the invasisity of bacterium and amount of plasmid transfer by 1000 and 10 fold, respectively. *In vivo*, BLG production was detected in isolated enterocytes after oral administration of LL-mInlA+BLG and was slightly higher than oral administration of LL-BLG.

## Results

### Mutated internalin A is produced on the surface of recombinant L. lactis strain

**Figure 1:**
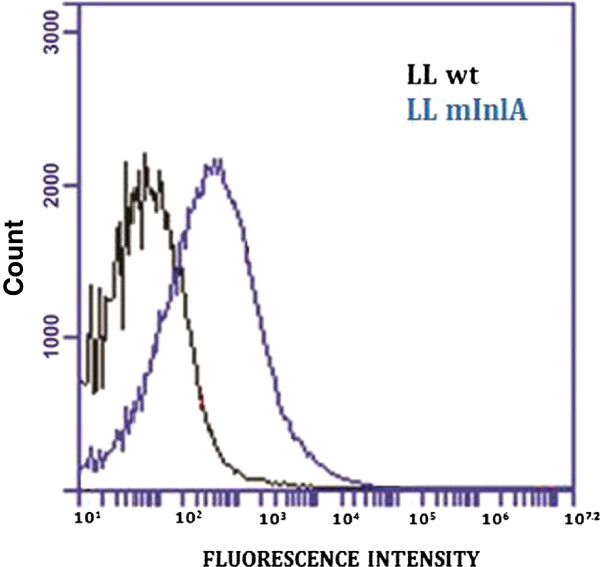
**Characterization of mInlA production at the surface of *L. lactis. *** Black peak corresponds to the negative control, the wild type strain (LL) and the blue peak corresponds to *L *. *lactis * strain producing mInlA (LL-mInlA+).

To investigate surface expression and production of mInlA, *L*. *lactis* NZ9000 and LL-mInlA+ strains were incubated with specific anti-mInlA monoclonal antibody and then with FITC-conjugated anti-Mouse IgG. Stained cells were analyzed by flow cytometry. As shown in Figure 1, LL-mInlA+ strain (blue peak) showed a significant shift in the fluorescence intensity comparing to the NZ9000 strain (black peak). No shift was observed when strains were incubated with FITC-labeled anti-Mouse IgG alone (data not shown). This experiment confirmed expression of mInlA on the surface of *L*. *lactis*.

### L. lactis producing mInlA is efficiently internalized by Caco-2 cells

**Figure 2:**
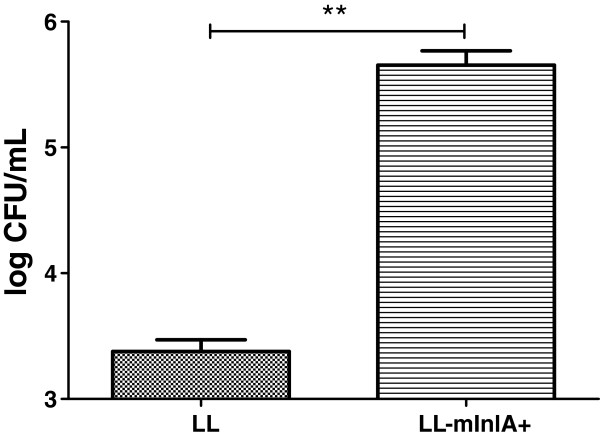
**Evaluation of the LL-mInlA+ invasiveness capacity in non-confluent Caco-2 cells. ** Caco-2 cells were co-incubated with NZ9000 and LL-mInlA+ strains during 1 h and then treated with gentamicin for 2 h. Cells were lysed and the number of CFU internalized was measured by plating. **, survival rates were significantly different (One-way ANOVA, Bonferroni’s multiple comparison test, p < 0.05). Results are means standard deviations of three different experiments, each time done in triplicate.

Non-confluent Caco-2 cells were incubated for 1 h with either NZ9000 or with LL-mInlA+. Non internalized bacteria were killed by gentamicin and intracellular bacteria enumerated after lysis of the eukaryotic cells. The LL-mInlA+ strain exhibited 1000-fold greater invasion rate than NZ9000 strain (Figure 2).

### LL-mInlA+ internalization analyzed by confocal microscopy

**Figure 3:**
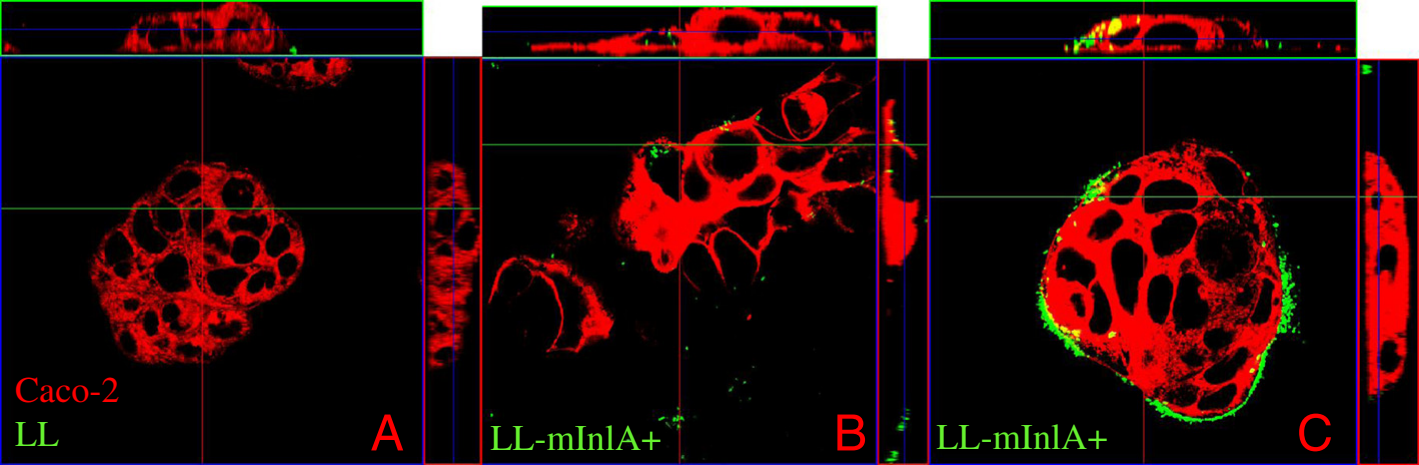
**LL-mInlA+ internalization in Caco-2 cells analyzed by confocal microscopy. ** NZ9000 and *L*. *lactis* producing mutated internalin A (LL-mInlA+) were stained with CFSE dye (in green) and co-incubated with Caco-2 cells. Cell membranes were stained with DiI cell-labeling solution (in red) and the fluorescent samples were analyzed by confocal microscopy as described in the methods. 3**A**. Non-internalization of NZ9000 strain in Caco-2 cells. 3**B**. Intracellular localization of LL-mInlA+ in some cells. 3**C**. LL-mInlA+ bind to the membrane of cell clusters where mInlA receptor, E-cadherin, is exposed.

LL-mInlA+ and NZ9000 strains were labeled with CFSE dye and then incubated with Caco-2 cells for 1 h. Cells were fixed and confocal images were obtained. Very few cell-associated bacteria could be detected after co-incubation with NZ9000 (Figure 3A). In contrast, the LL-mInlA+ strain strongly bound to the membrane of cell clusters which is compatible with the known binding of InlA to E-cadherin, a cell-cell adhesion molecule. In addition, LL-mInlA+ was located intracellularly in some cells (Figure 3C and B).

### LL-mInlA+ can efficiently deliver *in vitro* a DNA vaccine containing β-lactoglobulin cDNA

To test the ability of LL-mInlA+ to deliver a DNA vaccine plasmid *in vitro* to IECs, we transformed LL-mInlA+ strain with pValac:BLG [32], a plasmid derived from pValac [23] containing the cDNA for BLG, under the control of an eukaryotic promoter to generate strain LL-mInlA+BLG (Table 1).

**Table 1:** Bacterial strains and plasmids used in this work

**Strain/plasmid**	**Relevant characteristics**	**Source/reference**
**Bacterial strains**		
NZ9000	A derivative of *L*. *lactis* MG1363 wild type strain generated by the integration of the *NisRK* genes	45
LL	*L*. *lactis MG1363* containing pOri23 plasmid	40
LL-mInlA+	*L*. *lactis* NZ9000 strain containing pOri253:mInlA	This work
LL-BLG	*L*. *lactis* MG1363 strain containing pOri23 and pValac: BLG plasmid	32
LLmInlA+BLG	*L*. *lactis* NZ9000 strain expressing mInlA gene and carrying pValac: BLG plasmid	This work
**Plasmids**		
pPL2:mInlA	*E*. *coli* vector containing mInlA gene	30
pOri253Link	*L*. *lactis*-*E*. *coli* shuttle vector, Eryr	This work
pOri23	*L*. *lactis*-*E*. *coli* shuttle vector, Eryr	40
pValac: BLG	*L*. *lactis*-*E*. *coli* shuttle vector carrying the BLG gene under the control of the eukaryotic promoter IE CMV, Cmr	32
pOri253:mInlA	*L*. *lactis*-*E*. *coli* shuttle vector carrying the mInlA gene under the control of the constitutive PrfA promoter protein and harboring the native cell wall anchoring signal	This work

*Eryr* Erythromycin resistant; *Cmr* Chloramphenicol resistant.

**Figure 4:**
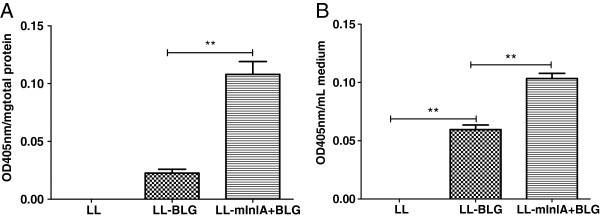
**BLG production in Caco-2 cells after co-incubation with LL-mInlA+BLG or LL-BLG. ** Caco-2 cells were co-incubated with LL, LL-BLG or LL-mInlA+BLG during 3 h. BLG was assayed 72 h after co-incubation in cellular protein extracts **(A)** or medium **(B)**. The results are expressed as mean ± SE values. Statistical significance between the groups was calculated using the One Way ANOVA followed by the “Bonferroni” post-test. Values of p < 0.05 were considered significant.

In order to monitor plasmid transfer and production of BLG in Caco-2 cells extracts, non-confluent Caco-2 cells were incubated with noninvasive *L*. *lactis* strains, LL and LL-BLG (see Table 1), or with LL-mInlA+BLG for three hours. After incubation with these bacteria, cell supernatant and proteins extracts from Caco-2 cells were tested for BLG expression using an EIA. BLG production was measured in Caco-2 cells protein extracts incubated with either LL-BLG or LL-mInlA+BLG. However, incubation with the LL-mInlA+BLG strain resulted in 10 fold higher levels of BLG compared to LL-BLG strain demonstrating that surface expression of mInlA enhanced intracellular delivery of the DNA vaccine DNA (Figure 4A).

Secreted levels of BLG were increased 2 fold after co-incubation with LL-mInlA+BLG compared to LL-BLG (Figure 4B). These data shows that LL and LL-mInlA+, can mediate gene transfer of a DNA vaccine to Caco-2 cells *in vitro* and that invasiveness significantly increases the efficiency of DNA delivery.

### DNA delivery efficiency *in vivo* is slightly improved by the production of mInlA

**Figure 5:**
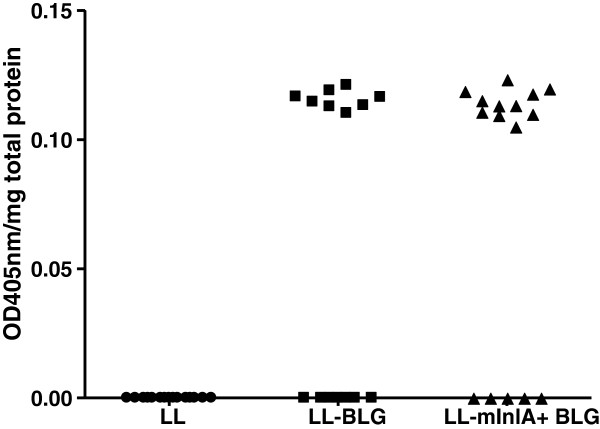
**β-Lactoglobulin detection in mice isolated enterocytes after oral administration of noninvasive and invasive lactococci strains.** Mice were orally administered 3 consecutive days with LL, LL-BLG or LL-mInlA+BLG. Seventy two hours after the last gavage, mice were sacrificed and BLG was assayed in protein extracts from isolated small intestine enterocytes. Results showed the sum of two independent experiments.

Mice were intragastrically administrated with LL, LL-BLG or LL-mInlA+BLG for three consecutive days, and the small intestine removed for isolation of IECs. BLG production was detected in protein extracts from IECs of mice administered with LL-BLG and LL-mInlA+BLG but not with control mice (Figure 5). In both of the LL-BLG and LL-mInlA+BLG treated groups, some mice did not show production of BLG suggesting that DNA delivery may be a stochastic event depending on environmental factors. Even if this trend was not statistically significant, the number of mice producing BLG (in each of the three individual experiments) was systematically higher (11 mice) in the group administered with invasive bacteria than with noninvasive bacteria (8 mice producing BLG) suggesting that the LL-mInlA+strain is a slightly better DNA delivery vehicle than non-invasive strain.

## Discussion

There is a large body of research demonstrating that the use of *L*. *lactis* is able to elicit humoral and cellular immune responses to an antigen produced in rodents (for reviews see [19-22]).

Recently, we showed the ability of either native or recombinant invasive *L*. *lactis* as both *in vitro* and *in vivo* DNA delivery vehicle [24-27]. Recombinant invasive *L*. *lactis* strains were obtained by producing heterologous invasins which are proteins expressed at the surface of pathogens responsible for their invasivity. We first built lactococci expressing Internalin A (InlA) from *Listeria monocytogenes* (LL-InlA+) and showed that LL-InlA+ were able to 1) deliver a plasmid *in vitro* and 2) be invasive *in vitro* and *in vivo* in guinea pigs [24]. Nevertheless, the use of LL-InlA+ is restricted because InlA does not bind efficiently to its murine receptor, the E-cadherin [33]. Subsequently, we produced another invasin, the Fibronectin Binding Protein A (FnBPA) from *Staphylococcus aureus* and demonstrated that LL-FnBPA+ were invasive and able to transfer a plasmid *in vitro* more efficiently than non-invasive *L*. *lactis*[25]. However, FnBPA requires an adequate local concentration of fibronectin in order to bind to its receptors, integrins [28,29], and this limitation could be a problem *in vivo*. So, in this study we produced a mutated Internalin A (mInlA) at the surface of *L*. *lactis*. The two mutations introduced were demonstrated to allow the binding of mInlA to murine E-cadherin thus permitting *in vivo* experiments with conventional mice [30,31].

We first checked that mInlA was expressed and properly directed to the surface of *L*. *lactis*. The shift of fluorescence peak obtained for LL-mInlA+ in FACS analysis was significantly higher as compared to NZ9000 strain thus confirming successful surface expression of mInlA on *L*. *lactis*. Other invasins, from Gram-positive bacteria, such as InlA or FnBPA, have already been successfully expressed in *L*. *lactis* confirming that the signal peptide for secretion and the anchoring signal are well recognized by the *L*. *lactis* machinery. Production of invasins from Gram-negative bacteria, such as *Yersinia pseudotuberculosis* invasin at the surface of *L*. *lactis* has never been successful (Denis Mariat, personal communication).

The invasivity was assessed by gentamicin assay in non-differentiated E-cadherin expressing human epithelial cell line Caco-2 cells. This experiment showed that LL-mInlA+strain is 1000-fold more invasive than NZ9000 strain. Wollert and collaborators (2007) observed a 2-foldincrease in the adhesion and invasion efficiency of *L*. *monocytogenes* strain producing mInlA compared to wild-type listeria expressing native InlA by using gentamicin-protection-invasion assays in Caco-2 cells [30]. A confocal image taken after gentamicin assay showed clearly that LL-mInlA+ is capable of adhering to and entering in non-differentiated Caco-2 cells. The preferential distribution of recombinant bacteria at the periphery of the Caco-2 cell islets can be explained by the fact that E-cadherin is accessible only at the periphery. A similar type of bacterial distribution, around the Caco-2 cell islets, was previously observed when Caco-2 cells were co-incubated with LL-FnBPA+[25].

LL-mInlA+ and LL strains were then transformed with pValac: BLG plasmid, co-incubated with Caco-2 cells and BLG expression was followed 72 h later by ELISA. BLG was detected in the cytoplasmic fraction of Caco-2 cells which were co-incubated with noninvasive and invasive strains carrying pValac: BLG. This data confirms prior observations that even noninvasive *L*. *lactis* can transfer functional plasmids to Caco-2 cells [23]. Cells were also capable of secreting the allergen, which is an interesting characteristic facilitating antigen uptake and presentation by professional APCs through cross-priming pathways [1]. The use of LL-mInlA+ improved BLG expression around ten times compared to noninvasive strain. Our hypothesis is that invasive lactococci can enter in higher numbers inside epithelial cells and thus deliver more plasmids.

Noninvasive and invasive *L*. *lactis*, carrying pValac: BLG or not, were orally administered for 3 consecutive days in BALB/c mice. On the fourth day, enterocytes from the small intestine were isolated and BLG production was measured by enzyme immunoassay (EIA). Isolated enterocytes from mice administered with invasive LL-mInlA+BLG produced the same amount of BLG as compared to mice immunized with noninvasive LL-BLG. Thus, we confirmed that noninvasive lactococci are able to transfer a functional plasmid *in vivo* in mice [27]. The use of LL-mInlA+BLG enhanced slightly the number of mice positive for plasmid transfer. Surprisingly, BLG production was not increased.

These results partly confirmed what we published recently with LL-FnBPA+BLG *in vitro* and *in vivo*[32]. Oral administration in mice of LL-FnBPA+BLG or LL-FnBPA+GFP elicited a GFP or BLG production in enterocytes. As with LL-mInlA+ the BLG production was not increased with LL-FnBPA+. However the number of mice producing BLG was significantly higher after oral administration of LL-FnBPA+BLG compared to non invasive LL-BLG. Considering these results it seems that LL-FnBPA+strain is a better DNA delivery vehicle than LL-mInLA+.

As no significant advantages were observed by using LL-mInlA+BLG compared to LL-BLG, we hypothesize that interactions of recombinant mInlA with their receptors were impeded in mouse intestinal epithelium. This lack of invasion *in vivo* was also observed by another group working with *E*. *coli* strain expressing invasin from *Yersinia pseudotuberculosis* as an oral vaccine for cancer immunotherapy. They showed that invasive *E*. *coli* was unable to enter gut epithelial cells due to a basolateral localization of the receptor, B1-integrin [34]. They demonstrated that invasive *E*. *coli* expressing *Y*. *pseudotuberculosis* invasin were selectively uptaken from the intestinal lumen into Peyer’s patches using an *ex vivo* model. Similarly, E-cadherin, the mInlA receptor, is also expressed on the basolateral membrane of IECs which are strongly linked to each other in the gut making E-cadherin less available. It has been shown recently that *L*. *monocytogenes* could enter the epithelial membrane through extruding epithelial cells at the top of the villi but mainly through goblet cells which are located deeper in the crypt [35]. It is thus possible that LL-mInlA+BLG strain is not able to reach its receptor deeply buried in the crypt. The pathway whereby bacteria could penetrate gut epithelial monolayers could be through Microfold (M) cells in Peyer’s patches. These cells are able to take up particles/bacteria from the lumen [36]. Nevertheless, we cannot exclude any possibility that lactococci can also interact with other cells from the epithelial membrane such as dendritic cells. Some subset of dendritic cells is now well known to produce dendrites, able to reach the lumen in order to sample its content [37].

The other hypothesis is that the plasmid would be released in the lumen by lysed lactococci and then captured by the enterocytes. It has been shown that lactococci do not persist in the gut and are very sensitive to its physico-chemical condition [38]. Most likely, plasmid transfer *in vivo* is a combination of both mechanisms, bacteria and released plasmid captures. Considering these data, the use of lactobacilli which persist longer in the gut than lactococci could be a better option for DNA delivery.

## Conclusions

Mutated Internalin A protein was successfully expressed at the surface of *L*. *lactis* NZ9000, as demonstrated by FACS analysis. LL-mInlA+ strain was demonstrated to be 1000 times more invasive as compared to NZ9000 strain. This invasiveness capacity was confirmed by confocal microscopy experiments wherein LL-mInlA+ was found to be attached to Caco-2 cells and intracellularly located. Assays of BLG detection after BLG expression by eukaryotic cells revealed that the invasive status improved plasmid transfer *in vitro*. *In vivo*, the number of mice expressing BLG was higher (n = 11) in the group immunized with invasive bacteria than with noninvasive bacteria (n = 8). Even though this difference was not statistically significant, these study suggests that LL-mInlA+ strain can be used as a DNA delivery vehicle for *in vitro* or *in vivo* experiments. The use of other LAB species which can persist longer in the gastrointestinal tract, such as lactobacilli, to mediate DNA transfer is currently being evaluated.

## Methods

### DNA manipulation and plasmids construction

Procedures for DNA manipulation were carried out as described by Sambrook et al. (1989) [39], with a few modifications. Plasmids were purified by the alkaline lysis method after bacterial incubation for 30 min at 37°C in TES solution (25% sucrose, 1 mM EDTA, 50 mM Tris–HCl pH 8) containing lysozyme (10 mg/ml). The quality of the DNA, including its concentration and purity, was estimated by measuring the absorbance at 260 nm and 280 nm in spectrophotometer (SpectraFluor Plus, Tecan). Restriction and modification endonucleases were used according to recommendations of the suppliers. Details concerning the plasmids used in this study are found in Table 1.

In order to construct pOri253Link:mInlA, mInlA gene was excised from pPL2:mInlA vector (9438 bp) [30] with BamHI and NotI restriction enzymes and gel purified generating a 3000 bp DNA fragment. pOri253Link plasmid (5857 bp) was derived from pOri253 [40] by modifying the multiple cloning site. Two complementary oligos CCGGGGGATCCTCGAGACGCGTCCATGGCGGCCGCTGCA and CCCTAGGAGCTCTGCGCAGGTACCGCCGGCG introducing the following restriction sites, BamhI, XhoI, MluI, NcoI and NotI were annealed and ligated into pOri253 previously digested with XmaI and PstI (underlined). BamHI/NotI-digested and purified pOri253Link and mInlA fragments were ligated using T4 DNA ligase (Invitrogen) to obtain pOri253:mInlA vector (9175 bp) (Table 1). Finally, pOri253:mInlA was transformed in *E*. *coli* DH5α and in *L*. *lactis* NZ9000 strain as described in the next section.

### Bacterial strains, media and growth conditions

Bacterial strains are listed in Table 1. Briefly, *L*. *lactis* NZ9000 strain were grown in M17 medium containing 0.5% glucose (GM17) at 30°C without agitation and 10 μg/ml of erythromycin (Ery) or 5 μg/ml of chloramphenicol (Cm) were added, when required. Electroporation of *L*. *lactis* NZ9000 with pOri253:mInlA and/or pValac: BLG [32] plasmids was performed as described by Langella et al. (1993) [41]. Transformants were plated on GM17 agar plates containing Ery or Cm at the same concentration mentioned above and incubated at 30°C for two days before subsequent freezing or colony forming unit (CFU) counting. Positive clones were confirmed by colony PCR using specific oligos.

### Mice handling

Specific pathogen-free BALB/c mice (females, 6 weeks of age; Janvier, France) were maintained under normal husbandry conditions in the animal facilities of the National Institute of Agricultural Research (UEAR, INRA, Jouy-en-Josas, France). All animal experiments began after allowing the animals 1 week for acclimation and were performed according to European Community rules of animal care and with authorization 78-149 of the French Veterinary Services.

### Detection of mInlA expression by *L. lactis* using flow cytometry analysis

*L*. *lactis* NZ9000 and recombinant *L*. *lactis* expressing mInlA were centrifuged (5000 rpm), washed with phosphate buffered saline (PBS) and then resuspended at a concentration of approximately 1x10^9^ CFU/ml in 500 μl of PBS containing 0.5% of bovine serum albumin (BSA) and 10 μg/mL of monoclonal antibody anti-InlA kindly provided by Dr. Pascale Cossart (Cell Biology and Infection Department/Unité des Interactions Bactéries-Cellules, Pasteur Institute, Paris). After one hour incubation at 4°C, the bacteria were pelleted by centrifugation washed with PBS and then resuspended in 500 μl of PBS plus 0.5% of BSA containing fluorescein isothiocyanate (FITC)-conjugated AffiniPure Fab fragment Goat Anti-Mouse IgG (H+L) (Jackson Immuno Research). After 1 h incubation at 4°C, bacteria were washed once more with PBS and fixed in 2% paraformaldehyde for 30 min at 4°C. FITC labeled antibody binding to InlA was assessed by flow cytometry (Accuri C6 Flow Cytometer®) using excitation at 494 nm and emission in the range of 510-530 nm (FL1-A channel). Data analysis was performed using CFlow Software (Accuri Cytometers, Inc.). The result was expressed as the average of three independent experiments performed in triplicate.

### Invasion assay of bacteria into intestinal epithelial cells

The human intestinal epithelial cell line Caco-2 (ATCC number HTB37) derived from a colon carcinoma was used to measure invasion capacity of each strain. Caco-2 cells were cultured in RPMI medium containing 2 mM L-glutamine (BioWhittaker, Cambrex Bio Science, Verviers, Belgium) and 10% fetal calf serum in p-24 plates (Corning Glass Works) until they reached 70-80% confluence. In the assays on non-confluent Caco-2 cells, approximately 4x10^5^ cells were present in each p-24 well. Bacterial strains were grown to an OD_600_ of 0.9–1.0, pelleted and washed in PBS, then added to the Caco-2 cell cultures at a multiplicity of infection (MOI) of approximately 1000 bacteria per eukaryotic cell. The gentamicin survival assay was used to evaluate bacteria survival. In summary, recombinant or wild type *L*. *lactis* were applied in the apical side of eukaryotic cells and co-incubated during one hour at 37°C, in 5% CO_2_. After this period, cells were washed in order to remove bacteria in excess and then 150 μg/mL of gentamicin was added for 2 h to kill the extracellular bacteria. Cells were then lysed with 0.2% triton-X 100 diluted in water. Finally, serial dilutions of the cell lysate were plated for bacterial counting. CFU of intracellular bacteria were expressed as the average of three independent gentamicin assays performed in triplicate. Invasion rate was calculated as the ratio of CFU counts.

### Confocal laser scanning

Bacteria were stained as described by Lee et al. (2004) [42]. Stationary phase culture of recombinant or wild type *L*. *lactis*, were washed twice in PBS and stained with 50 μM of green fluorescent dye carboxyfluorescein succinimidyl ester (CFSE) at 37°C for 20 min under constant shaking in the dark. CFSE labeled bacteria were used to perform the invasion assay as described above in non-differentiated Caco-2 cells grown on filter inserts. After 1 h of infection, cells were washed three times with PBS and fixed using 4% paraformaldehyde. Cell membranes were stained with 1 μM Vybrant® CM-DiI cell-labeling solution (Invitrogen) for 1 h at room temperature. Cells were mounted in Vectashield solution (Vector Labs, Burlingame, USA) to minimize photobleaching. Confocal images were obtained using a Zeiss LSM 510 system consisting of a Zeiss Axioskop with a Zeiss Plan Neofluar 63x NA 1.3 oil objectives. Stacks of images were reconstructed using Zeiss LSM software.

### β-Lactoglobulin (BLG) expression by human intestinal epithelial cells after incubation with bacteria

In order to measure BLG expression and secretion by human epithelial cells the gentamicin survival assay was performed with Caco-2 cells as described above, however, bacteria and Caco-2 cells were incubated for three hours. After gentamicin treatment, plates were maintained for 72 h at 37°C, in 5% CO_2_. Supernatant was collected by centrifugation at 78.2 g (800 rpm) for 10 min and stored at -80°C. One mL of PBS supplemented with a cocktail of protease inhibitors (Roche) was then homogenized by sonication (3 times 10 s). Samples were kept at -80°C and used to measure BLG production using an Enzyme ImmunoAssay (EIA) described in the next section.

### Enzyme immunometric assay (EIA) for quantification of bovine β lactoglobulin in human epithelial cells

The method used for BLG quantification is described elsewhere [43]. In summary, 96 microtitre plates were coated with 3.5 μg/ml of anti-BLG monoclonal antibody, diluted in 50 mM phosphate buffer (PB) pH 7.4, and incubated overnight at room temperature. After washing, plates were blocked with EIA buffer (0.1 M PB pH 7.4; 1 g/1 L BSA; 0.15 M NaCl; 0.001 M Na_2_EDTA; 0.1 g/1 L sodium azide) and stored sealed at 4°C until use. Standard (recombinant BLG) and samples diluted in EIA buffer were added and kept at 4°C for 18 h. After this time, plates were extensively washed and then acetylcholinesterase conjugated monoclonal anti-BLG antibody (1 Ellman Unit/ml) was added for 18 h at 4°C. After washing, Ellman reagent was added and enzymatic reaction was measured at 405 nm in a spectrophotometer (SpectraFluor Plus, Tecan).

### Oral administration of mice

Conventional BALB/c mice, 3 to 6 weeks of age were purchased from INRA animal care facilities (Jouy-en-Josas, France), acclimatized for 1 week before immunization under standard animal husbandry conditions in the animal facility (Unité d'Expérimentation Animale, Jouy-en-Josas, France). Mice (n = 8) were intragastrically administered with 1x10^9^ (CFU) of strains, LL, LL-BLG or LLmInlA-BLG on 3 consecutive days using a gavage tube feeding. On the fourth day, the small intestine was collected for subsequent BLG quantification in isolated IECs.

### Intestinal epithelial cells isolation

Mice were euthanized, and their small intestines were removed, rinsed with complete DMEM medium (containing 2 mM L-glutamine and 10% fetal calf serum). The length of intestine was opened and submerged in buffer A (in mM: 120 NaCl, 4.7 KCl, 2.4 KCl, 1.2 KH_2_PO_4_, 1.2 Na_2_HP0_4_, 25 NaHCO_3_, 10 HEPES, 5 EDTA, 0.5 DTT, 0.25% BSA; at pH 7.4 warmed to 37°C) for 20 min with agitation at 240 rpm [44]. Cells were collected by centrifugation (415.73 g – 2000 rpm – for 5 min) at room temperature, washed once with PBS and lysed by sonication (3 times, 10 s). The cell lysate was centrifuged for 10 min at 3143.98 g (5500 rpm), then the supernatant was recovered and stored at -80°C. The EIA to detect BLG was performed as described above.

### Statistical analyses

The results are expressed as mean ± standard error (SE) values. Statistical significance between the groups was calculated using the One Way ANOVA (and nonparametric) test, followed by the “Bonferroni” post-test. Values of p < 0.05 were considered significant.

## Competing interests

The authors declare that they have no competing interests.

## Authors’ contributions

The work presented here was carried out in collaboration between all authors. MA performed the main laboratory experiments and wrote the paper. JK helped with the confocal microscopy experiment and data analysis. FL constructed, provided pOri253:mInlA plasmid and initiated the project. PL, AM, and VA defined the research theme, helped to orient the work and revised the manuscript. JMC designed of the project, coordinated it, wrote and revised the manuscript. All authors have contributed to the writing of the paper and approved the final manuscript.
